# Fisetin Modulates Antioxidant Enzymes and Inflammatory Factors to Inhibit Aflatoxin-B1 Induced Hepatocellular Carcinoma in Rats

**DOI:** 10.1155/2016/1972793

**Published:** 2015-11-22

**Authors:** Brajesh Kumar Maurya, Surendra Kumar Trigun

**Affiliations:** Biochemistry Section, Department of Zoology, Banaras Hindu University, Varanasi 221005, India

## Abstract

Fisetin, a known antioxidant, has been found to be cytotoxic against certain cell lines. However, the mechanism by which it inhibits tumor growth *in vivo* remains unexplored. Recently, we have demonstrated that Aflatoxin-B1 (AFB1) induced hepatocarcinogenesis is associated with activation of oxidative stress-inflammatory pathway in rat liver. The present paper describes the effect of *in vivo* treatment with 20 mg/kg b.w. Fisetin on antioxidant enzymes *vis-a-vis* oxidative stress level and on the profile of certain proinflammatory cytokines in the hepatocellular carcinoma (HCC) induced by two doses of 1 mg/kg b.w. AFB1 i.p. in rats. The reduced levels of most of the antioxidant enzymes, coinciding with the enhanced level of reactive oxygen species in the HCC liver, were observed to regain their normal profiles due to Fisetin treatment. Also, Fisetin treatment could normalize the enhanced expression of TNF*α* and IL1*α*, the two proinflammatory cytokines, reported to be involved in HCC pathogenesis. These observations were consistent with the regression of neoplastic lesion and declined GST-pi (placental type glutathione-S-transferase) level, a HCC marker, in the liver of the Fisetin treated HCC rats. The findings suggest that Fisetin attenuates oxidative stress-inflammatory pathway of AFB1 induced hepatocarcinogenesis.

## 1. Introduction

In general, genotoxic agents are known to initiate neoplastic lesions by inducing DNA damage [1]. Aflatoxin-B1 (AFB1), produced by* A. flavus* and* A. parasiticus* fungi, is a genotoxic agent which causes hepatocellular carcinoma (HCC) by making AFB1-DNA adducts mainly in the liver cells [2]. This is because it is metabolized by the liver specific CYP450 (3A4) enzymes to produce highly reactive AFB1-8,9-epoxides that bind at N^7^ of guanine, thus creating lesions mainly in hepatocytes DNA [3]. Moreover, only those DNA aberrations drive hepatocytes to become tumorigenic which allow generation of tumor supportive microenvironment around [4].

Using diethylnitrosamine (DEN) induced HCC model, it has been described that the genotoxic damage of DNA is likely to induce oxidative stress to initiate hepatocytes necrosis resulting in release of the proinflammatory cytokines to drive HCC progression [5]. This process might implicate alterations in the cellular antioxidant enzymes of the cells undergoing genotoxic necrosis [6, 7]. The three antioxidant enzymes, superoxide dismutase (SOD), catalase, and glutathione peroxidase (GPx), mainly constitute antioxidant defense system of the cells [8, 9]. In particular, SOD1 (Cu-Zn SOD) is considered more relevant, as it catalyzes committed step of the antioxidant pathway [10] and has been reported to exist at a very low concentration in most of the growing tumors [11, 12]. Downstream to SOD1, catalase and GPx play important roles in removing H_2_O_2_ produced by SOD activity. GPx, in particular, is responsible not only for metabolizing H_2_O_2_ but also for maintaining rapid turnover of GSH, a critical cellular antioxidant. Importantly, modulations in GPx isoforms, GPx1 and GPx2, have been found associated with the tumor development [13, 14].

During the interplay of oxidative stress-inflammatory pathway, a number of proinflammatory cytokines have been identified to drive genotoxically affected hepatocytes to undergo compensatory proliferation [5, 15, 16]. TNF*α* has attracted much attention in this respect [17, 18]. IL1*α* is another member of inflammatory cascade which has been found to be associated with the tumor development and cancer cells metastasis [19, 20]. Some experimental data also suggest that prooxidative condition and inflammatory cytokines potentiate each other's effects in many ways during tumor progression. For example, enhanced TNF*α* level has been found to be associated with increased ROS generation by declining the level of SOD1 in U937 cells [21]. Similarly, downregulation of IL1*α* and IL1*β* in melanoma cell lines has been observed to normalize ROS level in those cells [22]. Thus, it is reasonable to examine modulation of oxidative stress-inflammatory pathway as a therapeutic target in AFB1 induced hepatocarcinogenesis.

Indeed, antioxidant enzymes have been found to serve as relevant pharmacological targets in a number of tumor models [11]. Modulation of all the key antioxidant enzymes by Emodin, an anthraquinone, in Dalton's lymphoma (DL), resulting in regression of the tumor* in vivo* [23], is a relevant example in this context. Certain exogenous antioxidants have been reported to prevent HCC development during DEN induced [24] and against AFB1 induced hepatocarcinogenesis as well [25].

Recently, we have reported that AFB1 toxicity declines all the antioxidant enzymes to activate oxidative stress-inflammatory pathway as main initiator of hepatocarcinogenesis in those rats [15]. This necessitated investigation on whether modulation of antioxidant enzymes and proinflammatory cytokines could serve as a therapeutic target to regress AFB1 induced HCC progression.

During the recent past, natural products have attracted much attention for their anticancer roles. Fisetin is a dietary polyphenol which was primarily predicted to serve as a strong ROS scavenger compound [26]. Indeed, by activating glutathione system and by scavenging cellular ROS, Fisetin has been described to prevent growth of the lung fibroblast cells [27]. However, besides its ROS scavenging ability, the data, derived mainly from* in vitro *studies, suggest that this compound shows cytotoxicity against a number of cell lines by modulating some of the tumor associated biochemical/molecular targets like inhibition of CDKs by downregulating NF*κ*B [28], inhibition of PI3K/Akt pathway in prostate cancer cells [29], and retarding angiogenic mechanisms in the endothelial cells [30].

Since Fisetin has been shown to induce apoptosis by downregulating bcl2 in the Huh7 cells (HCC cell line) as well [31], this compound deserves special merit to evaluate its anticancer activity against HCC* in vivo. * Though information is limited about* in vivo* anticancer activities of this compound, a report does indicate its role as a modulator of oxidative stress factors against benzopyrene induced lung carcinoma in mice [32]. We have also observed that AFB1 intoxication implicates alterations in the antioxidant enzymes and proinflammatory cytokines to develop HCC in rats [15] and that Fisetin treatment is able to normalize the level of GST-pi (glutathione-S-transferase, placental type), a HCC marker, and to regress HCC lesions in those HCC livers (data of the present report).

Therefore, the present study aimed to investigate whether a nontoxic dose of Fisetin is able to modulate the HCC growth supportive profiles of the antioxidant enzymes* vis-a-vis* oxidative stress markers and proinflammatory cytokines in AFB1 induced HCC rat liver.

## 2. Material and Methods

### 2.1. Chemicals

Fisetin (3,3′,4′,7-tetrahydroxyflavone) and* Aspergillus* extracted Aflatoxin-B1 were procured from Sigma-Aldrich, USA. SOD1 and GST-pi polyclonal antibody and primers for TNF*α* and IL1*α* were obtained from Santa Cruz Biotechnology, USA, and from Genetix, India, respectively. All other chemicals were purchased from Sisco Research Laboratory (SRL) Chemicals, India.

### 2.2. Animals

Male Charles foster rats (18–20 weeks old), procured from central animal house IMS-BHU, India, and maintained under controlled condition of temperature and humidity with alternate 12 h light/dark cycle, were used for this study. Rats were fed with standard pellet diet and water* ad libitum*. The study was conducted according to the ethical norms approved by the Institutional Animal Care and Use Committee (IACUC), Animal Ethical Committee of Banaras Hindu University, Varanasi (reference number Dean/10-11/169).

### 2.3. Experimental Protocol

Rats were randomly divided into three groups consisting of 5-6 rats each. Based upon the results of pilot experiments and as described in the previous report [15], the HCC group rats were administered intraperitoneally (ip) with two consecutive doses of 1.0 mg/Kg b.w. AFB1 dissolved in DMSO on the same day and monitored for development of HCC up to the 10th week. Fisetin treated HCC group (HCC + F) rats were administered with 20 mg/Kg b.w. Fisetin/day i.p. after the 6th week of AFB1 intoxication and this continued up to the 10th week. Based on pilot experiments and as reported in case of the mouse model [32], this dose of Fisetin was found to be nontoxic to the normal rats. The control group rats were similarly administered with DMSO throughout the treatment period.

At the end of the treatment period, rats from all the three groups were sacrificed by cervical dislocation after anesthesia (as per IACUC guidelines) and liver was excised. Livers from all the three groups were then subjected to histopathological and biochemical/molecular studies.

### 2.4. Liver Histology

Liver histology was performed as described earlier by our lab [15]. In brief, the liver sections were sliced into 0.3–0.5 cm pieces from control, HCC, and HCC + F groups rats. They were fixed in Bouin's fluid for 16–18 h and were transferred to 70% ethanol. This was followed by alcoholic dehydration and embedding in paraffin. Liver sections of 7 *μ*m thickness were cut and spread on the poly-L-lysine precoated slides. Slides were then subjected to Hematoxylin-Eosin (HE) staining. After mounting in DPX, slides were analyzed under Leica 2000 microscope.

### 2.5. Biochemical Analysis

#### 2.5.1. Extract Preparation

For native PAGE analysis, 10% liver extract was prepared in 0.2 mM Tris-HCl buffer (pH 7.4) and centrifuged at 10,000 ×g to collect partially enriched cytosolic fraction. For western blot analysis, 10% homogenate was prepared in 0.1 mM HEPES buffer (pH 7.4) containing 0.3 M KCl, 1 mM EDTA, 0.1% Triton X-100, 1 mM DTT, 0.001 mM PMSF, and 0.002 mM benzamidine and centrifuged at 10,000 ×g to collect partially enriched cytosolic fraction.

#### 2.5.2. Biochemical Estimations

*ROS*. NBT reduction assay was performed for ROS measurement in the liver extracts as reported previously [33]. Briefly, 2% liver extract, diluted in PBS, was added with the NBT-PBS (1 mg NBT/mL) solution in the ratio of 0.5 mL/mL and incubated at 37°C for 4 h. After centrifugation, the pellet was washed thrice with methanol and dissolved in 2.0 mL mix of 2 M KOH and DMSO. Absorbance was recorded at 630 nm. The OD obtained was compared with a standard plot constructed against NBT and values were expressed as mole of NBT/mg protein. 

*Total Glutathione*. Total glutathione was estimated in the liver extracts following a previously reported procedure [15]. Briefly, 0.1 mL liver extract was mixed with 1.5 mL of 0.2 M Tris buffer (pH 8.2) followed by addition of 0.1 mL of 0.01 M 5,5′-dithiobis(2-nitrobenzoic acid) (DTNB). The mixture was made 10 mL with methanol and incubated for 30 min. After centrifugation, absorbance of the supernatant was read at 412 nm and values were expressed as nM/mg protein.

Protein content was estimated following the method of Lowry et al., 1951 [34].

### 2.6. Native PAGE Analysis of SOD1, Catalase, and GPx

As described previously [15], for nondenaturing PAGE analysis of SOD1, CAT, and GPx, the extract containing 40 *μ*g protein was subjected to 8% nondenaturing PAGE followed by development of substrate specific achromatic bands against dark background for the specific antioxidant enzyme. Gels were scanned and the enzyme bands were quantified by the gel densitometry software Alpha Imager 2200.

### 2.7. Western Blot Analysis

Western blot analysis was performed as described previously [15]. Briefly, samples containing 60 *μ*g proteins were subjected to 10% SDS PAGE followed by transferring the protein bands to nitrocellulose membrane and probing them against anti-GSTpi and SOD1 antibody (1 : 1000 dilutions). The membrane was then probed with HRP conjugated secondary antibody (1 : 5000 dilutions). ECL SuperSignal West Pico kit was used to develop protein bands on X-ray films. HRP conjugated monoclonal *β*-actin antibody (1 : 10,000) was used as the loading control.

### 2.8. Semiquantitative RT-PCR

As described previously [15], total RNA was isolated from the liver tissue using TRI reagent following the protocol of the kit supplied from Sigma-Aldrich. Briefly, 2 *μ*g RNA sample was used for cDNA synthesis using random hexamer primer from the Revert Aid first strand cDNA synthesis kit (MBI Fermentas). Reaction mixture contained 19 *μ*L of Taq polymerase buffer, 0.2 mM dNTPs, 1 U of Taq polymerase, and 10 pmol of primer. The rat gene specific primers used were TNF*α* (forward: 5′-ACTCCCAGAAAAGCAAGCAA-3′; reverse: 5′-AGCAGGAATGAGAAGAGG-3′), IL1*α* (forward: 5′-AATCCTCTGAGCTTGCCAGG-3′; reverse: 5′-GAGGGCAAAAGACTGACCCA-3′), and *β*-actin (forward: 5′-TCTACAATGAGCTGCGTGTG-3′; reverse: 5′-AATGTCACGCACGATTTCCC-3′). Amplification products were analyzed by 2% agarose gel electrophoresis and visualized by ethidium bromide staining and quantified by the gel densitometry software Alpha Imager 2200.

### 2.9. Statistical Analysis

Experimental data was subjected to Student's *t*-test analysis for statistical significance. The probability values of less than 0.05 were considered statistically significant and results were presented as mean ± SD, where *n* = 4–6 for each group.

## 3. Results

### 3.1. Effect of Fisetin on Histopathology and HCC Marker

**Figure 1:**
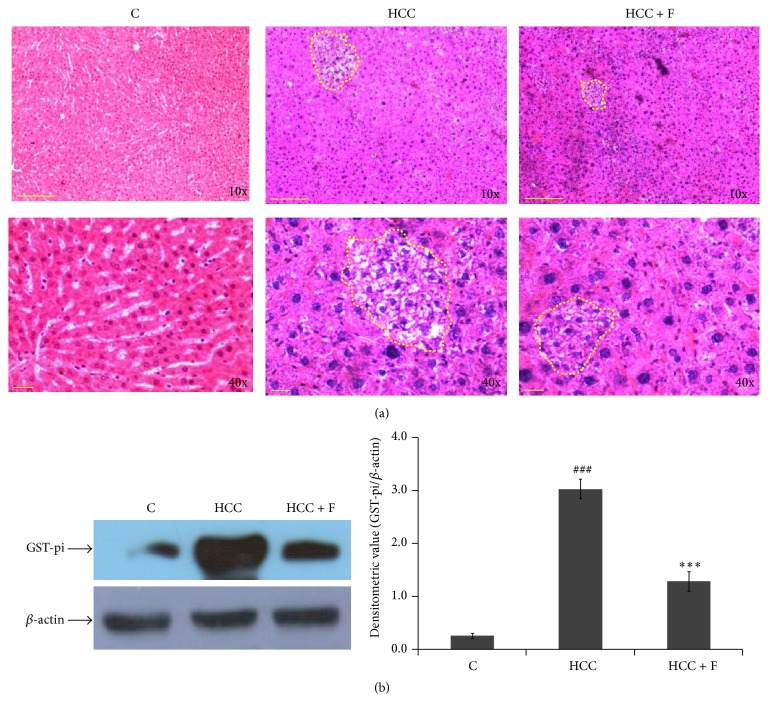
Effect of Fisetin on histopathology of HCC liver. Representative photomicrographs of liver sections from control (C), HCC, and HCC + F groups at 10 W stage have been shown. In (a), upper panel shows 10x magnification with scale bar of 200 *μ*m and lower panel shows 40x magnification with scale bar of 50 *μ*m. Dotted line encircles FAH area as a mark of neoplastic lesion. (b) shows level of GST-pi in the liver from control, HCC, and Fisetin treated HCC rats, wherein liver extract containing 60 *µ*g protein in each lane was subjected to 10% SDS-PAGE followed by western transfer and detection of GST-pi bands against a polyclonal anti-GST-pi. The photograph is representative of the three western blot repeats. Normalized densitometry values of GST-pi/*β*-actin have been presented as mean ± SD from three western repeats. ^###^ *p* < 0.001 (control versus HCC group) and ^*∗∗∗*^ *p* < 0.001 (HCC versus HCC + F group).

In general, appearance of foci of altered hepatocytes (FAH) regions, in histological preparations, is considered to represent neoplastic lesions during HCC development.  Figure 1(a) illustrates that, in comparison to the normal trabecular arrangement of hepatocytes seen in the liver section from the normal rats, regions of compressed trabeculae with compact hepatocytes, representing discrete FAH areas, could be seen in case of the liver from HCC group rats. Moreover, after Fisetin treatment, FAH regions are seen to be reduced remarkably with fewer number of compact hepatocytes within in the HCC liver.

Recently, GST-pi has been demonstrated to serve as a reproducible marker for AFB1 induced HCC progression. Therefore, to ascertain effect of Fisetin on HCC progression, the profile of GST-pi protein was also compared in the liver from the control, HCC, and Fisetin treated HCC group rats. As compared to the control group rats, ~4x increase (*p* < 0.001) in GST-pi level could be observed in the liver from the HCC group rats, which was brought back to its control level in the liver from the Fisetin treated HCC rats (Figure 1(b)).

### 3.2. Effect of Fisetin on Oxidative Stress and HCC Markers

**Figure 2:**
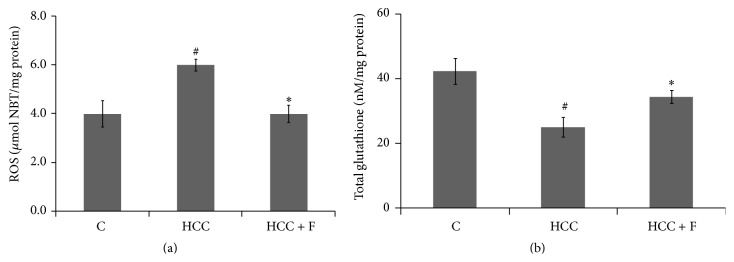
Effect of Fisetin on ROS (a) and glutathione (b) levels in the liver from control (C), HCC, and Fisetin treated HCC rats. Values have been represented as mean ± SD, where *n* = 6 and each experiment is done in triplicate. ^#^ *p* < 0.05 (control versus HCC group) and ^*∗*^ *p* < 0.05 (HCC versus HCC + F group).

Enhanced level of ROS is associated with AFB1 induced HCC progression. To ascertain whether Fisetin could suppress oxidative stress in AFB1 treated rats, ROS levels in the liver from control and experimental group rats were compared. A significant rise in ROS level (*p* < 0.05) was observed in the liver from the HCC rats as compared to the control counterparts. However, after Fisetin treatment, the enhanced ROS level in HCC liver was seen to be decreased significantly (*p* < 0.05) to regain its normal value (Figure 2(a)).

Enhanced level of glutathione, a nonenzymatic antioxidant, is considered critical for maintaining reducing equivalence in the cells facing oxidative stress. According to Figure 2(b), the liver from HCC group rats showed a significant decrease (*p* < 0.05) in glutathione level as compared to the liver from the control group rats. Moreover, such a decline of glutathione in the HCC liver could be recovered to its normal value in the HCC group rats administered with Fisetin.

### 3.3. Effect of Fisetin on the Profile of Antioxidant Enzymes: SOD, Catalase, and GPx

**Figure 3:**
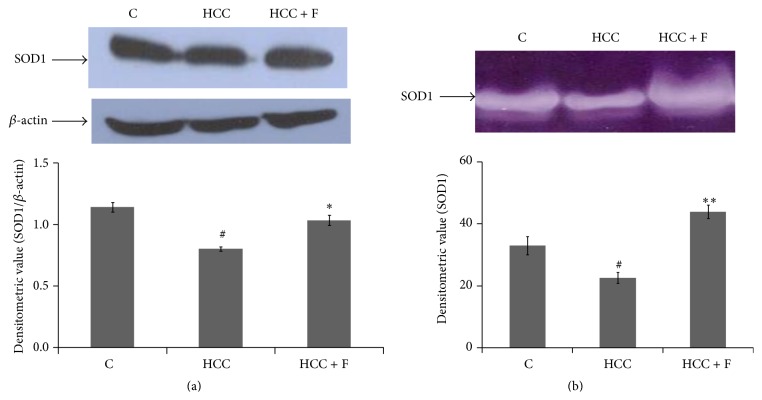
Effect of Fisetin on expression (a) and activity profile (b) of SOD1 in the liver from control (C), HCC, and Fisetin treated HCC rats. In (a), liver extract containing 60 *µ*g protein in each lane was subjected to 10% SDS-PAGE followed by western transfer and detection of SOD1 bands against a polyclonal anti-SOD1. The photograph is representative of the three western blot repeats. Normalized densitometry values of SOD1/*β*-actin have been presented as mean ± SD. (b) shows 8% nondenaturing PAGE results of 40 *µ*g protein loaded in each lane. After electrophoresis, gel was subjected to development of substrate specific SOD1 band. The gel photograph is representative of the four PAGE repeats. The relative densitometric values of SOD1 band have been presented as mean ± SD from the four PAGE repeats. ^#^ *p* < 0.05 (control versus HCC group) and ^*∗*^ *p* < 0.05, ^*∗∗*^ *p* < 0.01 (HCC versus HCC + F group).

Declined SOD1 level is often considered accountable for the rise of ROS at cellular level. In the present context, the expression and activity of SOD1 were measured in the liver from the control and the two experimental group rats. There was a significant decline (*p* < 0.05) in the expression of SOD1, as compared to the control group rats, in the liver from the HCC group rats (Figure 3(a)). This pattern of SOD1 expression was seen to be consistent with the activity profile of this enzyme in those livers (Figure 3(b)). Moreover, both the expression (Figure 3(a)) and activity of SOD1 (Figure 3(b)) could regain their normal values in the liver from the Fisetin treated HCC rats.

**Figure 4:**
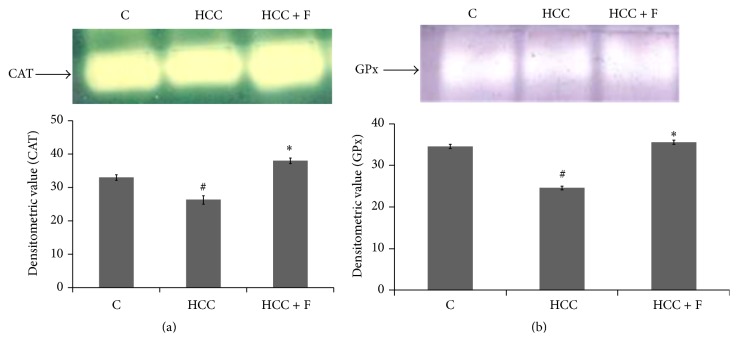
Effect of Fisetin on H_2_O_2_ metabolizing enzymes, catalase (a) and GPx (b), in the liver from control (C), HCC, and Fisetin treated HCC rats. The upper panels of (a) and (b) show 8% nondenaturing PAGE results of 40 *µ*g protein loaded in each lane. After electrophoresis, gels were subjected to development of substrate specific catalase and GPx bands, respectively. The gel photographs are representative of the four PAGE repeats. The relative densitometric values of catalase and GPx bands have been presented as mean ± SD from the four PAGE repeats. ^#^ *p* < 0.05 (control versus HCC group) and ^*∗*^ *p* < 0.05 (HCC versus HCC + F group).

SOD is the committed enzyme of the antioxidant pathway that neutralizes O_2_^−^ by converting them into H_2_O_2_. Catalase and GPx together metabolize H_2_O_2_ by utilizing GSH. Thus, catalase and GPx both play important roles in maintaining ROS homeostasis in the cells. According to Figures 4(a) and 4(b), the active levels of both of these enzymes were found to be declined significantly (*p* < 0.05) in the HCC liver as compared to the liver from the control group rats. However, after the treatment with Fisetin, the profiles of both catalase and GPx were found to be enhanced significantly to finally regain their values around the control liver.

### 3.4. Effect of Fisetin on Inflammatory Cytokines (TNF*α* and IL1*α*)

**Figure 5:**
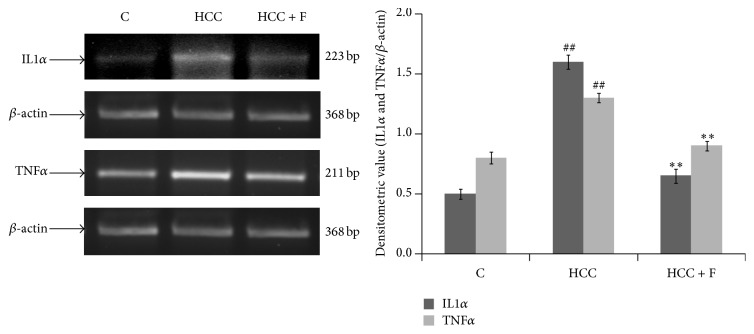
Effect of Fisetin on the level of proinflammatory cytokines, TNF*α* and IL1*α*, in the liver from control (C), HCC, and Fisetin treated HCC rats. The figure shows representative RT-PCR photographs from four repeats with the normalized densitometric values of TNF*α*/*β*-actin and IL1*α*/*β*-actin as mean ± SD from four RT-PCR repeats. ^##^ *p* < 0.01 (control versus HCC group) and ^*∗∗*^ *p* < 0.01 (HCC versus HCC + F group).

It has been demonstrated that AFB1 intoxication enhances TNF*α* and IL1*α* levels to finally support oxidative stress-inflammatory pathway of hepatocarcinogenesis. Since we observed that Fisetin administration is able to normalize oxidative stress parameters (Figures 2–4) in the HCC liver, it was speculated that such a biochemical change may diminish upregulated profile of TNF*α* and IL1*α* as well. Indeed, Figure 5 illustrates that the enhanced levels of TNF*α* and IL1*α* mRNA in HCC liver (*p* < 0.05) could be recovered to the values observed in case of the normal liver due to the Fisetin treatment to the HCC group rats.

## 4. Discussion

With regard to tumor development, oxidative stress is now evident to act as a double edged sword; some amount of oxidative stress stimulates tumor growth [35]; however, persistently enhanced oxidative stress, generated mainly due to depleted endogenous antioxidant system, has been demonstrated to induce apoptosis in the tumor cells* in vitro* [36] and* in vivo* as well [37, 38]. As such, this mechanism, although it needs to be confirmed in case of a higher number of* in vivo* tumor models, provides a biochemical basis to design therapy targeted to modulate antioxidant system in the tumor cells. In a recent report from this lab, it has been demonstrated that AFB1 induced hepatocarcinogenesis also implicates oxidative stress imposed due to decrease in the levels of all the antioxidant enzymes [15]. Moreover, it was interesting to observe that Fisetin, a dietary flavonol, is able to bring down the enhanced level of GST-pi, a HCC marker (Figure 1(b)), and could reduce the neoplastic lesions (FAH areas) in the HCC liver significantly (Figure 1(a)). In general, both GST-pi level and appearance of FAH regions are considered end point parameters to ascertain genotoxin induced HCC development in rat models [5, 15] and, thus, it was argued that Fisetin is able to regress AFB1 induced HCC in rats. However, since this pattern was consistent with the reversal of the enhanced ROS level in those HCC livers (Figure 2(a)), attempt was made to explore whether Fisetin regresses HCC by modulating antioxidant enzymes in the HCC liver* in vivo*.

Structurally, due to possession of three OH groups around, Fisetin is primarily known as a potent free radical scavenger [39]. In the present context, however, Fisetin was found not only to normalize ROS level but also to regain the level of depleted glutathione in the HCC liver (Figure 2(b)). This hinted at the multimodal action of Fisetin towards maintaining antioxidant milieu in the HCC cells. Indeed, some* in vitro* studies suggest that this compound is able to modulate different tumorigenic factors [27] including endogenous antioxidant factors at cellular level [30].

Mainly, the three antioxidant enzymes, SOD, catalase, and GPx, constitute central antioxidant mechanism to prevent oxidative insult at cellular level. SOD is the first enzyme of the antioxidant pathway that catalyzes dismutation of O_2_^−^ into a less toxic H_2_O_2_ compound [12]. Out of the two main SOD isoforms, SOD1 and SOD2, the level of SOD1 is found to be more critical in ROS mediated cancer progression [11]. It has been reported that reduced level of SOD1 facilitates genotoxin induced tumorigenesis via maintaining a high level of O_2_^−^ whereas increased SOD1 level has been demonstrated to attenuate this process [40, 41]. In the present context also, the HCC associated low level of SOD1 and consequently increased level of ROS in the AFB1 induced HCC liver could be recovered to their respective normal levels due to the treatment with Fisetin (Figures 3(a) and 3(b)).

Although H_2_O_2_, produced by SOD1, is considered relatively less toxic, its rise has been found to support the genotoxin induced tumorigenesis including AFB1 induced HCC as well [15, 42]. Therefore, its degradation by the two downstream enzymes, catalase and GPx, becomes equally critical for preventing oxidative stress induced tumorigenesis. It has been demonstrated that enhanced level of GPx alone can prevent oxidative insult in SOD1 and catalase dual suppressed cells, thereby suggesting concordant roles of these three antioxidant enzymes in tumor progression/regression [43, 44]. Since declined levels of both GPx and catalase, reported in case of AFB1 induced HCC [15], are recovered back to their normal levels due to the Fisetin treatment (Figures 4(b) and 4(a)), it may be discerned that Fisetin modulates all the three antioxidant enzymes to prevent rise in tumor supportive ROS level in the HCC liver.

In addition to preventing a rise in ROS level, GPx activity contributes to maintaining the level of reducing equivalents in the form of glutathione in the cell [45, 46]. This is because GPx catalyzed reaction involves glutathione turnover in the cells [47]. A relative decrease in glutathione level has also been found to be associated with the rise in ROS level during genotoxins induced tumorigenesis, which could be prevented due to the exogenous glutathione administration [48]. We could also observe that AFB1 induced HCC progression is accompanied with the declined glutathione level in the AFB1 induced HCC liver [15]. However, its level in those HCC livers was recovered due to the Fisetin treatment (Figure 1(b)).

Taken together, the findings of Figures 3 and 4 suggest that Fisetin modulates concordantly all the enzymes of antioxidant pathway which could account for prevention of ROS mediated HCC progression in the AFB1 treated rats. Though information is scanty about alterations in antioxidant enzymes by Fisetin in* in vivo* tumor models, it has been described that this compound does modulate oxidative factors against benzopyrene induced lung carcinoma in mice [32].

There could be more than one mechanism by which oxidative stress drives a cell towards neoplastic progression. Moreover, in case of genotoxin induced tumorigenesis, implication of oxidative stress-inflammatory pathway has been found to be the most plausible one [23, 49]. Using DEN induced HCC model, it has been speculated that oxidative stress is likely to induce local hepatocytes necrosis, thereby secreting certain proinflammatory cytokines around, which ultimately drives the neighboring cells to undergo compensatory proliferation and thus HCC progression [5]. Indeed, this pathway has recently been found to drive AFB1 intoxicated hepatocytes towards HCC progression as well [15]. Therefore, it was reasonable to examine whether Fisetin is able to modulate HCC associated inflammatory factors in the AFB1 induced HCC liver.

Among the inflammatory cytokines, TNF*α* has been given much attention because its deficiency has been demonstrated to prevent formation of neoplastic lesions during DEN induced hepatocarcinogenesis [17, 18]. IL1*α* is another cytokine which has been found to be implicated in the oxidative stress led necrotic death and consequently tumor progression [5]. Concordant with the declined levels of all the antioxidant enzymes, both of these cytokines have also been found to be overexpressed in the AFB1 induced HCC liver [15]. Not much information is available to derive a mechanistic link for reciprocal changes between antioxidant enzymes and the inflammatory factors; in a human cell line, it has been reported that enhanced TNF*α* represses SOD1 promoter activity via JNK/AP-1 signaling pathway [21]. It is reported here that Fisetin treatment could decline the enhanced level of both TNF*α* and IL1*α* (Figure 5), which is consistent with recovery in SOD1 activity (Figure 3), declined ROS level (Figure 2(a)), and HCC regression (Figure 1). Thus, it is argued that this compound could normalize proinflammatory-antioxidant pathway by declining TNF*α* expression and thus preventing SOD1 depletion in the HCC liver.

## 5. Conclusion

Targeting tumor growth associated biochemical events by nontoxic compounds is an evolving concept in cancer chemotherapy. In case of genotoxicity mediated tumorigenesis, imposition of oxidative stress is considered critical for driving a normal cell to undergo neoplastic progression* in vivo*. This paper describes that a nontoxic dose (examined on normal rats) of Fisetin, a natural flavanol, is able to normalize ROS led inflammatory pathway of AFB1 induced hepatocarcinogenesis in rats. And it does so by modulating the enzymes of the main antioxidant pathway in the HCC cells. The findings suggest that modulating antioxidant enzymes and proinflammatory factors could be the relevant mechanisms to inhibit tumor development* in vivo*.
